# Association of Type D personality and mild cognitive impairment in patients with hypertension

**DOI:** 10.3389/fpsyg.2022.974430

**Published:** 2022-11-16

**Authors:** Qingfang Ye, Li Liu, Yini Wang, Ling Li, Zhengjun Wang, Guojie Liu, Ping Lin, Qiujie Li

**Affiliations:** ^1^College of Nursing of Harbin Medical University, The Second Affiliated Hospital of Harbin Medical University, Harbin, China; ^2^Department of Basic Nursing, School of Nursing, Harbin Medical University, Daqing, China; ^3^Department of Cardiology, The Second Affiliated Hospital of Harbin Medical University, Harbin, China

**Keywords:** hypertension, mild cognitive impairment, Type D personality, medication adherence, negative affectivity

## Abstract

**Objective:**

The aim of this study was to evaluate the association between Type D personality and mild cognitive impairment (MCI) in patients with hypertension.

**Methods:**

A total of 324 subjects with hypertension were included in the study. All of them completed questionnaires on demographic characteristics, Type D personality Scale, Montreal Cognitive Assessment (MoCA), Beck Anxiety Inventory (BAI) and Beck Depression Inventory (BDI). The Type D personality effect was analyzed as both dichotomous and continuous methods.

**Results:**

The incidence of MCI was 56.5% in hypertensive individuals. Type D personality presenting as a dichotomous construct was an independent risk factor of MCI (odds ratio [OR] = 2.814, 95% confidence interval [CI] = 1.577–5.021, *p* < 0.001), after adjusting for ages, sex and some clinical factors. Meanwhile, main effect of negative affectivity component was independently related to the prevalence of MCI (OR = 1.087, 95%CI = 1.014–1.165, *p* = 0.019). However, associations between the main effect of social inhibition component (OR = 1.011, 95%CI = 0.924–1.107, *p* = 0.811) as well as the interaction of negative affectivity and social inhibition (OR = 1.013, 95%CI = 0.996–1.030, *p* = 0.127) with MCI were not found.

**Conclusion:**

The findings suggest that Type D personality is strongly associated with MCI in patients with hypertension. The negative affectivity component of the Type D appears to drive the correlations between Type D and MCI. These findings provide new ideas for studying the mechanisms underlying the relationship between personality and cognitive decline in hypertensive individuals.

## Introduction

Hypertension is a growing public health problem worldwide. Currently, it is estimated that there are 290 million hypertension patients 18 years and older in China (Wang et al., 2018). Hypertension not only damages target organs such as the heart, brain and kidneys, but can also cause impairment in cognitive function. Additionally, the latest evidence suggests that hypertension may speed cognitive decline, regardless of age (de Menezes et al., 2021). Mild cognitive impairment (MCI), described a transitional stage between age-related cognitive decline and dementia (Petersen et al., 2014), is common in patients with Hypertension (Qin et al., 2021). Early identification and prevention of MCI is essential in hypertension individuals, as these patients are more likely to progress into dementia in the following years (Busse et al., 2006; Birns and Kalra, 2009). Thus, it is one of great significance to understand the risk factors associated for MCI in order to reduce the risk of dementia in patients with hypertension (Kim et al., 2013). Studies of traditional risk factors, such as age, education, income, diabetes and dyslipidemia, have helped identify subgroups of hypertensive individuals who are at risk of cognitive impairment (Heizhati et al., 2021; Paradela et al., 2021; Yamamoto et al., 2022). However, the psychosocial risk factors have received less attention, especially the personality traits.

Recently, accumulating evidence showing that personality traits may be key predictors of cognitive function (Sutin et al., 2019; Weinstein et al., 2019). Type D personality (‘distressed personality’) trait, described as a tendency to experience higher levels of negative affectivity (NA) and social inhibition (SI), has been linked to poor outcomes in patients with hypertension and cardiovascular disease (Oliva et al., 2016; Su and He, 2019). European cardiovascular prevention guidelines have included Type D personality as a psychosocial risk marker (Piepoli et al., 2018). Previous studies have shown that Five Factor Model (FFM) personality traits were associated with the risk of MCI (Ayers et al., 2020). Both dimensions of Type D show substantial relationships to main dimensions of five-factor model, with NA significantly associated to neuroticism and SI to introversion (Kupper and Denollet, 2007). To the best of our knowledge, no research have explored the directly relationship of Type D personality with MCI in hypertensive individuals.

The potential deleterious effects of Type D personality on MCI are likely associated with inflammatory reaction. Recent studies have shown that neuroinflammation plays a pivotal role in the development of MCI in hypertensive individuals (Yang and Zhou, 2019). Patients with Type D personality were shown to have elevated levels of inflammatory factors including C-reactive protein, tumor necrosis factor-α (TNF-α) and interleukin-6 (Conraads et al., 2006), the expressions of these cytokines associated with inflammatory-cell infiltrates can increases β-amyloid burden, and eventually lead to cognitive dysfunction (Rosenberg, 2005). Furthermore, Type D personality has also been associated with negative health-related behavior of patients, such as smoking cigarettes, consuming alcohol (Ginting et al., 2016), poor medication adherence (Li et al., 2020), and unhealthy dietary habits (Wang et al., 2020). The results of previous studies confirmed that these factors can increase the risk of MCI (Yan et al., 2022).

Despite these well-known correlations, the effect of Type D personality on MCI is poorly understood. The current study sought to evaluate the association between Type D and MCI in hypertensive individuals. We attempted to demonstrate that hypertensive individuals with Type D have a higher incidence of MCI than do non–Type D hypertensive individuals.

## Materials and methods

### Patients and procedures

This cross-sectional study recruited 353 patients from the hypertension unit of the Second Affiliated Hospital of Harbin Medical University between May and December in 2021. Hypertension was defined as blood pressure (BP) ≥140/90 mm Hg at 3 different times or the use of antihypertensive medications. Patients were eligible for participation in the study if they were: (1) age ≥40 years old; (2) able to read and speak Chinese. Exclusion criteria are: (1) Secondary hypertension; (2) The scores of CDR-CV > 0.5; (3) co-existing with other diseases that may cause cognitive impairment (Parkinson’s, Alzheimer’s, vascular dementia, and demyelination of the central nervous system.); (4) co-existing with other serious systemic disease or tumor. We excluded 12 patients due to lack of complete laboratory indicators, 3 patients receiving a diagnosis of secondary hypertension after admission and 6 were diagnosed of dementia, 8 patients who did not complete the questionnaires. A total of 324 hypertensive individuals were included in the final analysis.

After gaining the patients’ informed consent, the general demographic questionnaire, Type D Personality Scale and Montreal Cognitive Assessment were evaluated by using a face-to-face format and each questionnaire evaluation took around 25 min. Highly qualified surveyors provided participants with in-depth explanations of the questionnaire, and offered necessary assistance for subjects to complete the surveys successfully. Meanwhile, clinical data were obtained from their medical documents and, if required, verified by consultant physician and subjects. This study was approved by The Harbin Medical University (China) Human Ethics Review Committee (ky2020-058), which was done in accordance with the Declaration of Helsinki.

### Measures

#### Type D personality

The Chinese translation The Type D Personality Scale (DS-14), developed in collaboration by Tilburg University in Netherlands, the Chinese University of Hong Kong, and the Institute of Psychology of the Chinese Academy of Sciences, was used to assess Type D personality (Denollet, 1998). This 14-item scale has two dimensions: negative affectivity (NA) and social inhibition (SI). The internal consistency and validity of the NA and SI were represented by Cronbach’s alpha value of 0.69 and 0.85, respectively (Cheng et al., 2018). Participants fill out each question with a score ranging from 0 to 4, and when both dimensions scored ten points or more, they are regarded to have type D personality. However, recently research revealed that Type D using the dichotomized structure may reduce the sensitivity of scale and generate spurious associations (Lodder et al., 2021).Thus, continuous scores for subscales NA and SI, as well as their main effects and statistical interaction were also studied in the study.

#### Medication adherence

Medication adherence was measured using the Morisky Medication Adherence Scale-8 (MMAS-8; Morisky et al., 2008). It is a self-report questionnaire consisting of 7 questions with binary answers and one question with a 5-point Likert scale. The total score of MMAS-8 ranges from 0 to 8 points, and higher total scores indicating higher adherence. The Cronbach’s alpha coefficients for the assessments of internal consistency and reliability of the MMAS-8 was 0.69.

#### Anxiety symptoms

Anxiety symptoms were assessed using 21-item Beck Anxiety Inventory (BAI) developed by A.T. Baker, which is a common self-assessment instrument for assessing symptoms of anxiety (Beck et al., 1988). Subjects were asked to rate the severity of each problem with a score ranging from 1 to 4 based on their experiences in the previous week. The sum of all items produced crude scores, and then converted into standard scores by the formula *Y*=Int (1.19*x*).

#### Depressive symptoms

Depressive symptoms were measured using Beck Depression Inventory**-**II (BDI-II). It was developed by A.T. Baker in 1996, which is one of the most commonly used self-report questionnaires with 21 items for assessing depressive symptoms (Beck et al., 1996). Subjects fill out each problem with a score ranging from 0 to 3 depending on their experiences. The sum of all items produced total scores of BDI ranged from 0 to 63, with higher scores associated with more severe depressive symptoms.

#### Non-dementia performance

The Chinese version of the Mattis Dementia Rating Scale (DRS-CV) was used for the exclusion of dementia. It comprised tests of memory, attention, construction, conceptualization, initiation and perseveration, and has been validated as a sensitive and effective tool for rating patients with dementia by many Chinese researchers (Chen et al., 2021). In the present study, a score of DRS-CV ≤0.5 was considered as non-dementia performance (Qian et al., 2021).

#### Montreal cognitive assessment

Cognitive status was measured using Montreal cognitive assessment (MoCA), which was compiled by Nasreddine et al. (2005), and revised by a Chinese researcher Lu et al. (2011). The scale consists of visuo-constructional skills, naming, attention and concentration, language, abstraction, recall and orientation, and has been validated as a sensitive and effective screening tool for MCI (Uchida et al., 2020). Moreover, Chinese expert consensus on diagnosis and treatment of senile hypertension complicated with cognitive impairment (2021 version) also recommend the MoCA as a screening tool for MCI in hypertensive patients (Hu and Wang, 2021).

Montreal Cognitive Assessment was done strictly face to face by trained investigators following the guidelines and was finished in 10–15 min. The sum of all items produced total scores of MoCA ranged from 0 to 30, and a score of MOCA < 26 was categorized as MCI. To correct for the bias caused by education attainment, one point was added to the total scores of those individuals with ≤12 years of education. Lower scores overall reflect the worse cognitive function of the individual.

#### General demographic and clinical data

The following general demographic and clinical data were collected: age, gender, education, body mass index (BMI), duration of illness, smoking (consumption of ≥1 cigarettes per day), continuous or cumulative ≥6 months (Engel et al., 1978), drinking [take alcohol more than 24 g a day (Glassman, 2010)], medical history related to hyperlipidemia, coronary heart disease (CHD) and diabetes mellitus. Laboratory indicators comprised fasting blood glucose, serum total cholesterol (TC), triglycerides (TG), high-density lipoprotein cholesterol (HDL-C), low density lipoprotein cholesterol (LDL-C) and white blood cell.

### Statistical analysis

All statistical analyses were done using SPSS software (version 26.0). Continuous variables with normal distribution are computed in terms of mean (standard deviation) and non-normally distributed variables are expressed as median (interquartile range). Categorical variables are presented as frequency and percentages. *t*-test was used to compare normally distributed continuous data and Mann–Whitney *U* test was used to compare non-normally distributed data. Categorical variables were compared using *χ*^2^-test. Multivariable analysis was employed to determine the relationship between Type D personality and MCI in hypertensive individuals. Identify covariates based on previous literature (Heizhati et al., 2021; Paradela et al., 2021; Yamamoto et al., 2022). Age and sex were entered as standard control variables. BMI, smoking history, duration of illness, CHD, blood glucose, LDL-C, medication adherence, BAI score and BDI score were used to see if Type D personality was an independent predictor of MCI.

Two distinct analytical approaches were used to explore the impact of Type D personality in our study. First, the Type D as a categorical variable was tested on the basis of cutoff score ≥10 on both subscales of the DS14. Then, we analyze the predictive power of Type D personality as a continuous variable, which were converted to *z*-scores for negative affectivity, social inhibition and their statistical interaction. *p* values < 0.05 were considered statistically significant.

## Results

### Type D personality and baseline characteristics

The average age of patients (*n* = 324) was 61.10 years, of whom 157 (48.5%) were women. A total of 113 patients (34.88%) were identified as individuals with Type D personality. There were no significant differences in demographic characteristics, the history of other diseases and medication adherence between type D and non-type D personality group. However, type D individuals had a significantly higher blood glucose (*z* = −2.186, *p* = 0.029), BAI score (*t* = −8.213, *p* < 0.001) and BDI score (*t* = −4.186, *p* < 0.001) than non-type D individuals (Table 1).

**Table 1:** Baseline characteristics of categorical Type D personality status (*n* = 324).

Variables	Total sample	Type D	Non-type D	Test values	*p* Value
	(*n* = 324)	(*n* = 113)	(*n* = 211)		
Age, *M* (SD)	61.10 (11.55)	61.97 (11.41)	61.87 (11.86)	*t* = −0.75	0.941
Female, *n* (%)	157 (48.45)	52(33.12)	105 (66.88)	*χ*^2^ = 0.413	0.52
BMI, *M* (SD)	24.78 (3.03)	24.58 (2.87)	25.14 (3.29)	*t* = −1.59	0.112
Duration of illness, *M* (SD)	8.55 (7.95)	8.27 (7.64)	9.08(8.51)	*t* = −0.873	0.383
CHD, *n* (%)	159 (49.07)	58 (36.47)	101 (63.52)	*χ*^2^ = 0.353	0.553
Hyperlipidemia, *n* (%)	72 (22.02)	24 (33.33)	48 (66.67)	*χ*^2^ = 0.097	0.755
Diabetes mellitus, *n* (%)	86 (26.54)	37 (43.02)	49 (56.98)	*χ*^2^ = 3.421	0.064
Smoking history, *n* (%)	89 (27.47)	38(42.70)	51(57.30)	*χ*^2^ = 3.304	0.069
Drinking history, *n* (%)	52 (16.05)	20 (38.46)	32 (61.54)	*χ*^2^ = 0.350	0.554
Laboratory indicators					
Blood glucose, M (IQR), mmol/l	5.47 (1.80)	5.70 (2.05)	5.40 (1.47)	*z* = −2.186	0.029
TC, M (IQR), mmol/l	4.12(1.43)	4.14 (1.47)	4.10 (1.36)	*z* = −0.366	0.714
TG, *M* (SD), mmol/l	1.75 (1.72)	1.69 (0.94)	1.79(1.20)	*t* = 0.691	0.09
HDL-C, *M* (SD), mmol/l	1.11 (0.35)	1.11 (0.38)	1.11 (0.34)	*t* = 0.067	0.947
LDL-C, *M* (SD), mmol/l	2.48(0.90)	2.58 (0.92)	2.43 (0.88)	*t* = −1.429	0.154
WBC, *M* (SD), mmol/l	6.81 (2.04)	7.09 (2.11)	6.65 (1.99)	*t* = −1.863	0.063
Medication adherence, *M* (SD)	6.59 (1.04)	6.54 (1.21)	6.62 (0.96)	*t* = −1.863	0.076
BAI score, *M* (SD)	40.17 (8.46)	44.97 (9.05)	37.59 (6.88)	*t* = −8.213	<0.001
BDI score, *M* (SD)	8.90 (5.51)	10.61 (6.66)	7.99 (4.54)	*t* = −4.186	<0.001

*M* (SD) = mean (standard deviation); BMI = body mass index; CHD = coronary heart disease; *M* (IQR) = median (interquartile range). TC = total cholesterol; TG = triglycerides. HDL-C = high-density lipoprotein cholesterol; LDL-C = low-density lipoprotein cholesterol; WBC = white blood cell; BAI = Beck Anxiety Inventory; BDI = Beck Depression Inventory.

### Type D personality and Montreal cognitive assessment

On MoCA, he mean total score for the study participants was 23.10 (SD-3.74), and about half of the patients were categorized to have MCI (56.48%). The incidence of MCI in patients with type D was significantly higher than patients without type D personality (*χ*^2^ = 20.329, *p* < 0.001). Moreover, When the patients with and without type D personality were compared for various domains of cognition as assessed by MoCA, those with Type D personality had significantly poorer functioning on the dimensions of visuo-construction (*t* = 2.849, *p* = 0.005), attention (*t* = 3.452, *p* = 0.001), language (*t* = 6.278, *p* < 0.001) and recall (*t* = 4.716, *p* < 0.001) (Table 2).

**Table 2:** Montreal cognitive assessment in Type D and non–Type D patients.

Variables	Total sample	Type D	Non-type D	Test values	*p* Value
	(*n* = 324)	(*n* = 113)	(*n* = 211)		
Qualitative variable					
MCI, *n* (%)	183 (56.48)	83 (45.35)	100 (54.65)	*χ*^2^ = 20.329	<0.001
Quantitative variable					
Visuo-construction, *M* (SD)	3.41 (1.13)	3.17 (1.18)	3.54 (1.09)	*t* = 2.849	0.005
Naming, *M* (SD)	2.72 (0.57)	2.65 (0.60)	2.75 (0.56)	*t* = 1.615	0.107
Attention, *M* (SD)	4.66 (1.11)	4.37 (1.17)	4.81 (1.04)	*t* = 3.452	0.001
Language, *M* (SD)	2.53 (0.62)	2.25 (0.66)	2. 68 (0.54)	*t* = 6.278	<0.001
Abstraction, *M* (SD)	1.43 (0.65)	1.35 (0.64)	1.47 (0.65)	*t* = 1.712	0.088
Recall, *M* (SD)	2.99 (0.84)	2.76 (0.84)	3.11 (0.82)	*t* = 3.619	<0.001
Orientation, *M* (SD)	5.36 (0.76)	5.26 (0.80)	5.42 (0.73)	*t* = 1.879	0.061
Total score, *M* (SD)	23.10 (3.74)	21.80 (3.59)	23.81 (3.63)	*t* = 4.766	<0.001

### Independent predictors of mild cognitive impairment

Table 3 shows the results of regression analysis using Type D personality as a dichotomized variable. The results showed that Type D personality was an independent predictor of MCI (OR = 2.814, 95% CI = 1.577–5.021, p < 0.001), after adjusting for conventional risk factors of cognitive dysfunction.

**Table 3:** Multivariate regression model for mild cognitive impairment with Type D personality categorized.

Variable	OR	95% CI		*p*
		Lower 2.5%	Upper 2.5%	
Type D personality	2.814	1.577	5.021	<0.001
Covariates				
Age	1.061	1.035	1.087	<0.001
Female	1.087	0.639	1.849	0.758
BMI	1.075	0.987	1.171	0.098
Smoking history	1.906	1.041	3.489	0.037
Duration of illness	1.01	0.974	1.048	0.583
CHD	1.303	0.798	2.127	0.291
Blood glucose	1.041	0.898	1.206	0.596
LDL-C	1.061	0.794	1.418	0.69
Medication adherence	0.8	0.643	0.995	0.045
BAI	0.997	0.962	1.034	0.871
BDI	1.047	0.992	1.105	0.093

OR = odds ratio; CI = confidence interval; BMI = body mass index; CHD = coronary heart disease; LDL-C = low-density lipoprotein cholesterol; BAI = Beck Anxiety Inventory; BDI = Beck Depression Inventory.

In Table 4, we present the multivariate regression model using Type D personality as a continuous variable. First, we conducted multivariate regression to see if the interaction of NA*SI (*z* scores) predicted MCI. The results indicate that the NA*SI (*z* scores) effects was non-significant (Model 1). Then, the NA*SI effects (*z* scores) was shifted out of the model to examine the main effects of NA and SI (Model 2), and the results showed that the main effect of NA (*z* scores) was an independent predictor of MCI (OR = 1.087, 95% CI = 1.014–1.165, *p* = 0.019).

**Table 4:** Multivariate regression model for mild cognitive impairment with NA by SI statistical interaction effect and main effects of NA and SI.

Variable	OR	95% CI		*p*
		Lower 2.5%	Upper 2.5%	
Model 1: NA by SI statistical interaction
NA (*z* scores)	1.075	1.001	1.153	0.046
SI (*z* scores)	1.022	0.933	1.12	0.637
NA*SI (*z* scores)	1.013	0.996	1.03	0.127
Covariates				
Age	1.059	1.033	1.085	<0.001
Female	1.038	0.611	1.764	0.89
BMI	1.079	0.99	1.177	0.085
Smoking history	1.902	1.04	3.479	0.037
Duration of illness	1.008	0.973	1.045	0.652
CHD	1.338	0.82	2.183	0.243
Blood glucose	1.047	0.901	1.215	0.55
LDL-C	1.08	0.805	1.45	0.606
Medication adherence	0.803	0.645	1	0.05
BAI	0.999	0.961	1.038	0.942
BDI	1.047	0.992	1.105	0.093
Model 2: NA by SI main effect
NA (*z* scores)	1.087	1.014	1.165	0.019
SI (*z* scores)	1.011	0.924	1.107	0.811
Covariates				
Age	1.059	1.033	1.085	<0.001
Female	1.029	0.606	1.746	0.917
BMI	1.073	0.984	1.169	0.11
Smoking history	1.963	1.076	3.581	0.028
Duration of illness	1.008	0.972	1.045	0.661
CHD	1.351	0.829	2.2	0.227
Blood glucose	1.055	0.909	1.224	0.483
LDL-C	1.09	0.813	1.462	0.564
Medication adherence	0.789	0.633	0.982	0.034
BAI	0.998	0.961	1.037	0.914
BDI	1.047	0.993	1.104	0.092

NA = negative affectivity; SI = social inhibition; OR = odds ratio; CI = confidence interval; BMI = body mass index; CHD = coronary heart disease; LDL-C = low-density lipoprotein cholesterol; BAI = Beck Anxiety Inventory; BDI = Beck Depression Inventory.

## Discussion

The present study sought to examine the association between Type D personality and MCI in hypertensive individuals, to our knowledge, this is the first study to investigate the influence of Type D personality on the incidence of MCI in the context of hypertension. As expected, we found that Type D personality was an independent risk factor for MCI in hypertensive individuals when it was analyzed as a dichotomous construct. The component of NA was significantly associated with MCI when the main effect of two dimensions and their interaction were analyzed. These findings show that the dimension of NA may play a role in the association between Type D personality and MCI.

The prevalence of MCI was 56.48% in our study population, which is significantly higher than the prevalence of MCI in Chinese adults (15.5%; Jia et al., 2020).Thus, it is necessary to understand the risk factors for MCI in hypertensive patients. It was suggested that conventional risk factors (such as age, smoking, duration of hypertension) are associated with MCI in patients with hypertension (Yamamoto et al., 2022). For instance, Muela et al. (2017) confirmed that age was independent predictors of MCI in patients with hypertension in different cognitive domains. Our results showed that age, smoking history and medication adherence were associated with MCI in patients with hypertension, which is consistent with the results of previous studies (Heizhati et al., 2021; Paradela et al., 2021; Yamamoto et al., 2022). Moreover, coronary heart disease, obesity, anxiety and depression can interfere with MCI (Heizhati et al., 2021; Yamamoto et al., 2022), however, these variables were not related to MCI in our study.

Although the incidence of MCI in hypertensive patients is associated with several influenced factors, psychological factor may be one of the most important triggers of it. In the present study, we used two different analytical methodologies to evaluate the association between Type D and MCI in hypertensive individuals. First, the multivariate regression model revealed that the MCI was influenced by Type D personality in a classified construct, even after adjustment for age, sex, and the risk variables of cognition impairment. Next, the interaction of NA and SI was analyzed as a continuous variable, and it was possible to get a null result. However, when we analyzed the main effects of the components, the model revealed that NA of Type D was linked to MCI. The findings suggest that NA component may be to blame for the links between Type D and MCI in hypertensive patients.

Emerging evidence has shown that Type D might be associated with increased inflammatory activation (Irwin and Vitiello, 2019). The levels of TNF-α, soluble TNF-α receptor 1 (sTNFR1) and soluble TNF-α receptor 2 (sTNFR2) were elevated in patients with Type D in previous cross-sectional research (Conraads et al., 2006). Furthermore, the prospective findings also confirmed that individuals with negative affect had higher levels of inflammatory markers, such as (TNF-α), interleukin-6 (IL-6) and interleukin-1β (IL-1β; Denollet et al., 2009). The changes of these inflammatory factors play an important role in the onset and progression of MCI (Hye et al., 2014). It is now widely accepted that systemic inflammation, including levels of TNF-α-related cytokines will damage connections of neurons and axons in the brain, and eventually lead to the development of cognitive dysfunction (Pillai et al., 2019). Also, the study from our project team have demonstrated that TNF-α, IL-6 and inflammation standardized sum scores mediated the relationship between the NA and plaque vulnerability in patients with coronary artery disease (CAD; Yi et al., 2022). As a result, inflammatory activation may be an underlying physiological mechanism that connects NA of Type D personality to MCI, and further study is needed to clarify.

Individuals with Type D personality are more likely to be exposed to a variety of stressors, which is another mechanism contributing to decreased cognitive performance in patients with hypertension. Prior investigators have shown that Type D patients would perceive higher severity of job pressure (Ogińska-Bulik, 2006), posttraumatic stress and social stress (Kunst et al., 2011) than non-type D counterparts. It is generally acknowledged that stress affects the central nervous system and can lead to cognitive problems (Johansson et al., 2013). In recent studies, it was reported that exposure to acute and chronic stress and may lead to poorer cognitive performance and worse memory function and declines in cognitive function over time (Aggarwal et al., 2014). A meta-analysis including 16 cohort researches showed that higher perceived stress was significantly associated with an increased risk of MCI, and it can increase the risk of MCI by 1.19 (Franks et al., 2021). Therefore, the vulnerability of individuals with Type D personality to acute and chronic stress supports the psychological plausibility that links Type D personality and increased MCI risk.

Previous research has demonstrated that scoring high on Type D personality had a tendency to experience unhealthy behaviors and poor medication adherence, both of which increased the risk of cognitive decline. Smoking, as one of the most popular adverse health habits has been linked to an increased risk of all-cause dementia” (Kim et al., 2013). In addition, recent research evidence suggests that elderly people who had quit smoking are less likely to develop MCI compared to those who smoke (Hu et al., 2019). In the present study, the results of our regression analysis also showed that smoking was significantly associated with MCI. Moreover, a number of reports have indicated that Type D personality is associated with lower medication adherence in patients with hypertension, coronary artery disease, and Type 2 Diabetes Mellitus (Consoli and Safar, 1988; Molloy et al., 2012; Oliva et al., 2016; Li et al., 2017). Some evidences suggest that adherence to antihypertensive medication is a critical factor to control blood pressure and prevent cognitive impairment (Okuno et al., 2001). Similarly, a study including 20,071 patients reported that a strong association between antihypertensive medication adherence and cognitive impairment in elderly hypertensive patients without dementia (Cho et al., 2018). Our statistical analysis indicated that medication adherence was an independent predictor of MCI in hypertensive individuals. However, there was no significant difference in medicine adherence between the Type D and non-Type D groups, which could be related to the small sample size of our study. Overall, these findings on behavioral pathways support the role of unhealthy behaviors and poor medication adherence as a possible mechanism that connects Type D to increased MCI risk.

In a word, personality is relatively unmodifiable, but there are physiological psychological, and behavioral pathways between Type D personality and MCI, and these mediating variables may be the targets of our interventions (Yi et al., 2022). As suggested by Denollet, the originator of Type D personality, interventions for psychological problems affecting the progress of somatic diseases should ideally concentrate on improving psychological function and decelerating disease progression, for which understanding of the psychobiological mechanisms is a prerequisite (Kupper et al., 2018). Thus, identifying pathways explaining the observed relationships between Type D personality and MCI in patients with hypertension is important to design precise and personalized interventions.

## Limitations of study

This study has several limitations. Firstly, it was conducted at a single center with a relatively small number of patients. Larger multi-center studies should be conducted to verify the effect of Type D personality on MCI. Secondly, this was a cross-sectional study, and we cannot infer the causal relationship between Type D personality and MCI. Finally, inclusion of other detailed neuropsychological measurements, such as the Big Five personality, anxiety and depression could have helped in better understanding of the impact of Type D personality on MCI.

## Conclusion

Our findings demonstrated that Type D personality as a dichotomous construct was an independent predictor of MCI in hypertensive individuals. Moreover, additional regression results revealed that the dimension of NA, but not the SI and NA*SI interaction, may drive the connection between Type D personality and MCI. As a result, these findings indicate the potential necessity of early identification of hypertensive patients with Type D personalities in order to identify those who are at higher risk of MCI. Early psychological intervention should be performed to reduce the incidence rate of MCI.

## Data availability statement

The original contributions presented in the study are included in the article/Supplementary material, further inquiries can be directed to the corresponding authors.

## Ethics statement

The studies involving human participants were reviewed and approved by The Harbin Medical University (China) Human Ethics Review Committee, The Harbin Medical University. The patients/participants provided their written informed consent to participate in this study.

## Author contributions

QY and LLiu contributed to investigation and wrote the original draft of the manuscript. YW and LLi contributed to the design of the study and revision of the manuscript. ZW and GL participated in the analysis and interpretation of data. PL and QL were responsible for conception and supervision of the project. All authors contributed to the article and approved the submitted version.

## Funding

This work was supported by the Fundamental Research Funds for the Provincial Universities (JFYQPY202103 and JFQN202103), the National Natural Science Foundation of China (72004045), China Postdoctoral Science Foundation (2021M701023).

## Conflict of interest

The authors declare that the research was conducted in the absence of any commercial or financial relationships that could be construed as a potential conflict of interest.

## Publisher’s note

All claims expressed in this article are solely those of the authors and do not necessarily represent those of their affiliated organizations, or those of the publisher, the editors and the reviewers. Any product that may be evaluated in this article, or claim that may be made by its manufacturer, is not guaranteed or endorsed by the publisher.
